# AIDS patients with Talaromycosis Marneffei exhibit inflammatory activation and depletion in their peripheral blood monocytes

**DOI:** 10.1371/journal.pntd.0014306

**Published:** 2026-05-04

**Authors:** Pengle Guo, Yingyin Yang, Xiejie Chen, Qingqing Li, Huihua Zhang, Xin Chen, Feilong Xu, Quanmin Li, Jingyi Ou, Xiaoping Tang, Linghua Li

**Affiliations:** 1 Infectious Disease Center, Guangzhou Eighth People’s Hospital, Guangzhou Medical University, Guangzhou, China; 2 Guangzhou Medical Research Institute of Infectious Diseases, Guangzhou, China; 3 Clinical Laboratory, Guangzhou Eighth People’s Hospital, Guangzhou Medical University, Guangzhou, China; 4 Institute of Infectious Diseases, Guangzhou Eighth People’s Hospital, Guangzhou Medical University, Guangzhou, China; Albert Einstein College of Medicine, UNITED STATES OF AMERICA

## Abstract

**Objectives:**

This study seeks to investigate the characteristics, dynamics, and association with disease progression of monocyte inflammation in patients with AIDS/Talaromycosis Marneffei (TSM). The findings are expected to contribute to a deeper understanding of the disease’s pathogenesis and prognostic factors, offering substantial potential for advancing diagnostic and therapeutic approaches and identifying new targets for immunotherapy.

**Methods:**

We conducted a cross-sectional study to compare individuals with AIDS/TSM to those with AIDS/Pneumocystis Pneumonia(PCP), AIDS/tuberculosis(TB), uncomplicated AIDS, and healthy controls by analyzing the ratios and absolute values of peripheral blood mononuclear cells. Correlation analyses were employed to elucidate the relationship between monocyte levels and disease progression and prognosis. Flow cytometry and transcriptome sequencing were utilized to characterize the monocytes in patients with AIDS/TSM. Subsequently, a prospective study was undertaken on the TSM cohort to observe changes in monocyte numbers and proportions.

**Results:**

We observed a significant reduction in both the number and proportion of these cells in TSM patients. This reduction was correlated with disease progression indicators such as platelet count (PLT), alanine aminotransferase (ALT), aspartate aminotransferase (AST), and albumin (ALB). Furthermore, the baseline absolute monocyte count was found to be a significant predictor of mortality. Utilizing flow cytometry to analyze monocyte subpopulations, we found that the decrease in monocytes in TSM patients was characterized by an increase in classical monocytes and a decrease in intermediate and non-classical monocytes. Transcriptome sequencing data of monocytes revealed that those in TSM patients exhibited inflammatory activation, which was more pronounced in patients who succumbed to the disease. Subsequently, we conducted a prospective study on the TSM cohort and observed that antifungal treatment led to a gradual normalization of monocyte numbers and proportions.

**Conclusions:**

The peripheral blood monocytes of patients with AIDS and TSM exhibit a significant reduction, which correlates with disease progression and prognosis. Furthermore, these mononuclear cells demonstrate signs of inflammatory activation alongside a quantitative depletion.

## Author summary

AIDS-associated Talaromycosis Marneffei (TSM) is a life-threatening opportunistic infection, and the role of monocyte inflammation in its pathogenesis and progression remains poorly understood. With the goal of deepening the understanding of the disease’s pathogenesis and prognostic factors, and providing new insights for the development of diagnostic markers, therapeutic strategies, and immunotherapy targets, this study aimed to investigate the characteristics, dynamic changes, and clinical relevance of monocyte inflammation in AIDS/TSM patients. To achieve this, we first conducted a cross-sectional study, comparing peripheral blood mononuclear cell (PBMC) parameters (ratios and absolute values) between AIDS/TSM patients and four control groups (AIDS/Pneumocystis Pneumonia [PCP] patients, AIDS/tuberculosis [TB] patients, uncomplicated AIDS patients, and healthy individuals). Correlation analyses were performed to explore the associations between monocyte levels and disease progression/prognostic indicators. Flow cytometry and transcriptome sequencing were further applied to characterize the phenotypic and molecular features of monocytes in AIDS/TSM patients. Additionally, a prospective follow-up study was carried out in the AIDS/TSM cohort to monitor the dynamic changes of monocyte counts and proportions during treatment. Our findings demonstrate that peripheral blood monocytes in AIDS/TSM patients undergo significant quantitative depletion, which is closely associated with disease progression and prognosis. Concurrently, these monocytes exhibit obvious inflammatory activation despite the quantitative reduction. These results highlight the critical role of monocytes in the pathophysiological process of AIDS/TSM, and suggest that monocyte-related parameters may serve as potential prognostic markers and therapeutic targets for this disease. The dynamic recovery of monocytes following antifungal treatment also provides evidence for the clinical value of monitoring monocyte changes in assessing treatment response.

## Introduction

Talaromyces marneffei (TM) is a significant opportunistic pathogenic fungus responsible for systemic disseminated infections, resulting in Talaromycosis Marneffei (TSM). This condition is predominantly prevalent in Southeast Asian countries and southern China, where it constitutes a major cause of hospitalization and mortality among AIDS patients [1,2]. Recent reports indicate that approximately 16% of hospitalized AIDS patients in Guangdong Province develop TSM, with the incidence rising annually [3]. Factors such as the growing number of immunocompromised individuals, global warming, and enhanced transportation are contributing to the expanding demographic and geographic distribution of TSM [4,5]. Despite the widespread availability of antiretroviral therapy (ART), the mortality rate for AIDS patients with TSM remains alarmingly high at 30% if effective antifungal treatment is not administered promptly [6,7]. The mononuclear macrophage system serves as a crucial defense mechanism against TM infection. In patients with AIDS, HIV infection results in a marked reduction in CD4 + T lymphocyte counts. This reduction is associated with a pronounced cellular immune deficiency, impairing the capacity of mononuclear macrophages to effectively engulf and digest TM, thereby facilitating disseminated infection. Despite the importance of this process, research on the immunological characteristics and alterations of monocytes remains limited. Investigating the immunological properties and changes in monocytes among AIDS patients with TSM (AIDS/TSM), both prior to and following antifungal therapy and antiretroviral therapy (ART), and examining their correlation with disease progression, holds significant potential for the understanding of the pathogenic mechanisms of TM in immunocompromised hosts, and also providing immunological markers with potential application value for clinical disease assessment and therapeutic efficacy monitoring.

## Materials and methods

### Ethics statement

This study was approved by the Ethics Review Board of the Guangzhou Eighth People’s Hospital (Approval No.202351288). Written informed consent was obtained from all the participants.

### Research subject

The participants were selected from hospitalized patients in the Infectious Department of Guangzhou Eighth People’s Hospital between January 2021 and December 2022.

TM Group: The study population comprised hospitalized patients with AIDS/TSM, admitted to the Infectious Diseases Department at the Guangzhou Eighth People’s Hospital, between January 2021 and December 2022. The inclusion criteria were as follows: I Age over 18 years; II Fulfillment of the diagnostic criteria for AIDS [8]; III Positive blood or bone marrow culture indicating TM infection; IV Provision of informed consent. Exclusion criteria included: I Presence of concurrent opportunistic infections such as Pneumocystis pneumonia (PCP) or tuberculosis (TB); II Presence of severe underlying conditions such as malignancies or cirrhosis; III Prior initiation of antifungal treatment upon admission.

PCP (Pneumocystis carinii pneumonia) group: The study population comprised AIDS/PCP inpatients admitted to the Infectious Diseases Department of the Guangzhou Eighth People’s Hospital between January 2021 and December 2022. Inclusion criteria for participants were as follows: I Age of 18 years or older; II Fulfillment of diagnostic criteria for AIDS [8]; III Evidence of PCP as indicated by bronchoalveolar lavage fluid microscopy or pathological examination; IV Provision of informed consent. Exclusion criteria included: I Presence of concurrent opportunistic infections, such as other fungal infections or tuberculosis; II Presence of severe underlying conditions, such as malignancies or cirrhosis; III Initiation of anti-PCP treatment prior to admission.

Tuberculosis (TB) Group: The study cohort comprised hospitalized patients diagnosed with AIDS and TB, admitted to the Infectious Diseases Department at the Guangzhou Eighth People’s Hospital, during the period from January 2021 to December 2022. The inclusion criteria for the study subjects were as follows: I Age of 18 years or older; II Fulfillment of the diagnostic criteria for AIDS [8]; III Confirmation of TB infection through acid-fast smear, culture, or molecular biology methods; IV Provision of informed consent. Exclusion criteria included: I Presence of other opportunistic infections; II Coexistence with severe underlying conditions such as malignancies or cirrhosis; III Initiation of anti-TB treatment prior to hospital admission.

AIDS group: Patients diagnosed exclusively with AIDS were chosen as the control group from outpatient follow-up records spanning January 2021 to December 2022. Inclusion criteria were as follows: I Confirmed diagnosis of HIV/AIDS; II Age of 18 years or older; III Not yet initiated on antiretroviral therapy (ART); IV Provision of signed informed consent; V CD4 count less than 50 cells/μL. Exclusion criteria included: I Presence of concurrent opportunistic infections; II Existence of significant underlying conditions such as malignancies or cirrhosis; III Initiation of ART prior to enrollment.

Healthy Control Group: Recruit a cohort of 20 healthy volunteers, systematically collect demographic information, and preserve peripheral blood samples for subsequent analysis.

### Data collection

Prior to enrollment, all study participants underwent a comprehensive evaluation and collection of clinical data, which included assessments of age, gender, CD4 + T cell count, CD8 + T cell count, HIV-1 viral load, complete blood count, biochemical parameters, coagulation time, and blood culture, among other measures.

### Study design

Initially, we conducted a cross-sectional study to compare patients with TM to those with Pneumocystis pneumonia (PCP), tuberculosis (TB), uncomplicated AIDS, and healthy individuals by analyzing the ratios and absolute values of peripheral blood mononuclear cells. Subsequently, correlation analyses were performed between the mononuclear cell values and clinical indicators, including platelet count (PLT), alanine aminotransferase (ALT), aspartate aminotransferase (AST), albumin (ALB), and disease severity. Monocyte subpopulations and the immune activation status were further analyzed using flow cytometry and RNA sequencing. Following this, we conducted a prospective study on the TM cohort to observe changes in monocyte numbers and proportions after antifungal treatment.

### Methods

#### Determination of the number and proportion of monocytes.

Using the Mindray 7500 fully automatic blood analyzer to count peripheral blood cells, the absolute value count (MONO, × 10^9^/L) and proportion of mononuclear cell blood unit particle concentration can be directly obtained.

#### PBMC isolation and flow cytometric analysis.

Peripheral blood mononuclear cells (PBMCs) were isolated from whole blood by Ficoll centrifugation and analyzed immediately or cryopreserved at -80°C in 80% fetal calf serum, 10% RPMI-1640 (Invitrogen, Grand Island, NY), and 10% dimethyl sulfoxide (DMSO) (Sigma–Aldrich, St.Louis, MO). DAPI (1 μg/ml) (Roche, Basel, Switzerland) was used to distinguish live cells from dead cells. The following anti-human antibodies were purchased from eBioscience (San Diego, CA): CD3-AF532 (clone:58-0038-42), CD14-APC-Cy7 (clone: 625620), CD16-BV605 (clone: 302040), CD11b-BV650 (clone: 301336) and their corresponding isotype antibodies. The anti-human CD3-PE-Cy7 was obtained from BD Biosciences (San Jose, CA). Cell phenotype was analyzed by flow cytometry on a flow cytometer (BD LSR II) (BD Biosciences). Data were acquired as the fraction of labeled cells within a live-cell gate set for 50000 events. For the flow cytometric sorting, BD Influx machine (BD Biosciences) was used. The strategy for classical, intermediate, and nonclassical monocytes, whose surface markers are: CD14++CD16-, CD14++CD16 + , CD14 + CD16 + . All experiments were performed in the biosafety laboratory.

#### CD14 monocyte RNA sequencing.

Using CD14 magnetic beads (Milton Biotec, Germany) to sort CD14 + monocytes from PBMCs, total RNA was extracted using an RNA extraction kit (Invitrogen, Grand Island, NY) and reverse transcribed into cDNA. The library was constructed using the VAHTS Universal V8 RNA seq Library Prep Kit for MGI library construction kit from Vazyme Biotech, China, and genome sequencing was performed.

### Statistical methods

In this study, statistical methods were employed to analyze the data. For datasets that met the assumptions of normal distribution and homogeneity of variance, results are presented as mean ± standard deviation (mean ± SD). Conversely, for datasets that did not meet these assumptions, results are expressed as median and interquartile range. Pairwise comparisons were conducted using the LSD-t test, non-parametric tests, or chi-square tests as appropriate. The correlation between different parameters was assessed using the Spearman rank test. Statistical analyses were performed using GraphPad Prism version 8.0a and SPSS version 25, with a significance threshold set at P < 0.05.

## Results

### 1. AIDS/TSM/TB/PCP and uncomplicated AIDS patients enrollment flowchart

**Fig 1:**
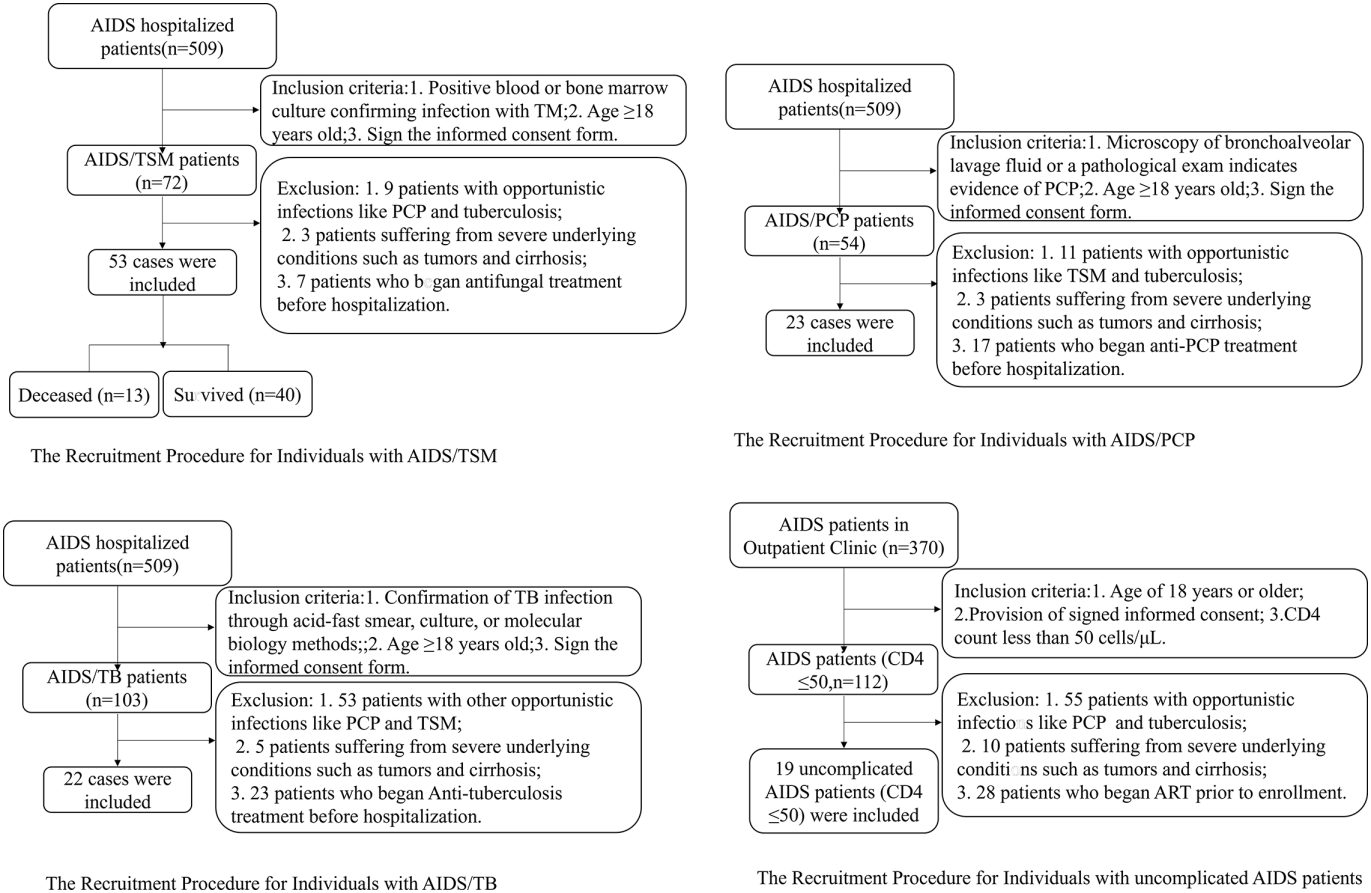
The Recruitment Procedure for Individuals with AIDS/TSM/TB/PCP and uncomplicated AIDS patients.

We selected a cohort of 53 AIDS/TSM patients from a total of 509 hospitalized AIDS patients at the Infectious Diseases Department of Guangzhou Eighth People’s Hospital, spanning the period from January 2021 to December 2022, based on predefined inclusion and exclusion criteria. The selection process is illustrated in Fig 1. For the control group, we concurrently identified 23 cases of AIDS/PCP, 22 cases of AIDS/TB, 19 cases of uncomplicated AIDS, and 20 healthy individuals, adhere to the specified inclusion and exclusion criteria.

### 2. The quantity of monocytes in patients with AIDS/TSM exhibited a significant reduction

**Fig 2:**
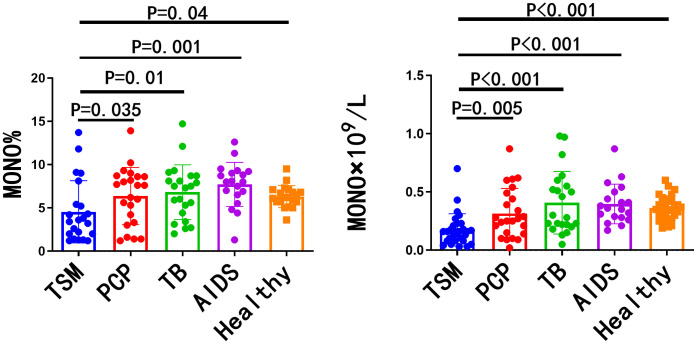
The quantity of monocytes in patients with AIDS/TSM exhibited a significant reduction. Blood routine tests were compared among AIDS/TSM, PCP, TB patients prior to treatment and the control group of AIDS and healthy people. A statistically significant reduction in the number of monocytes was observed. TSM: Talaromycosis Marneffei; PCP: Pneumocystis pneumonia; TB tuberculosis.

The study conducted a comparative analysis of blood routine tests from AIDS/TSM patients prior to antifungal treatment and a control group. The results revealed a statistically significant reduction in the number of monocytes, both in proportion and absolute value, in the AIDS/TSM cohort compared to the PCP, TB, AIDS, and healthy groups. These findings are illustrated in Fig 2.

### 3. The decline of monocytes in AIDS/TSM patients is closely related to disease progression

**Fig 3:**
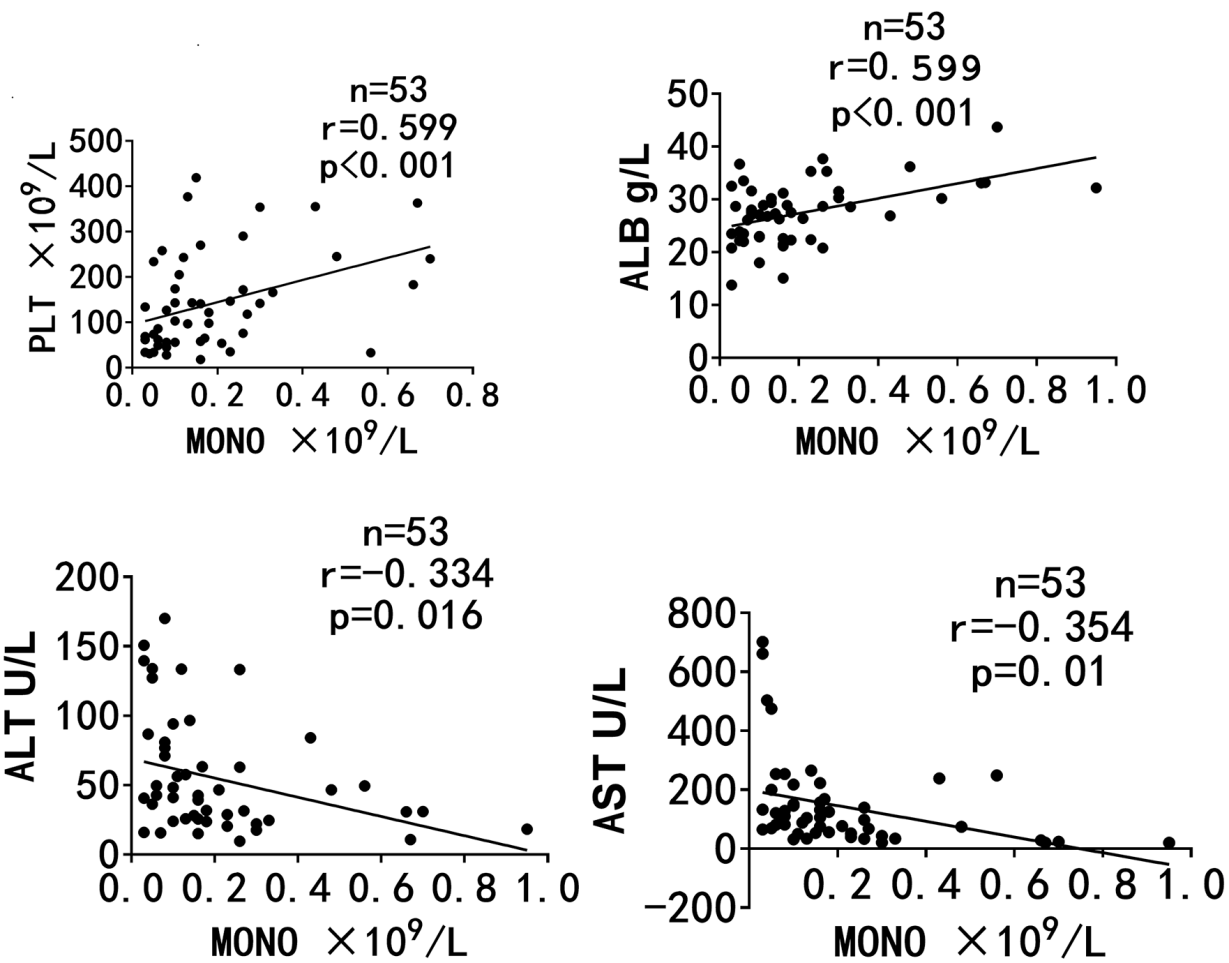
The decline of monocytes in AIDS/TSM patients is closely related to disease progression. A correlation analysis was performed to examine the relationship between monocyte counts and indicators of disease progression in these patients, such as platelet,albumin,alanine aminotransferase (ALT) and aspartate transaminase (AST) levels. The analysis revealed a positive correlation between monocyte counts and both platelet and albumin levels, while a negative correlation was observed with ALT and AST.

The baseline clinical indicators of AIDS/TSM Patients was shown in S1 Table. A correlation analysis was performed to examine the relationship between monocyte counts and indicators of disease progression in these patients. The analysis revealed a positive correlation between monocyte counts and both platelet and albumin levels, while a negative correlation was observed with alanine aminotransferase (ALT) and aspartate transaminase (AST) levels, as illustrated in Fig 3.

**Fig 4:**
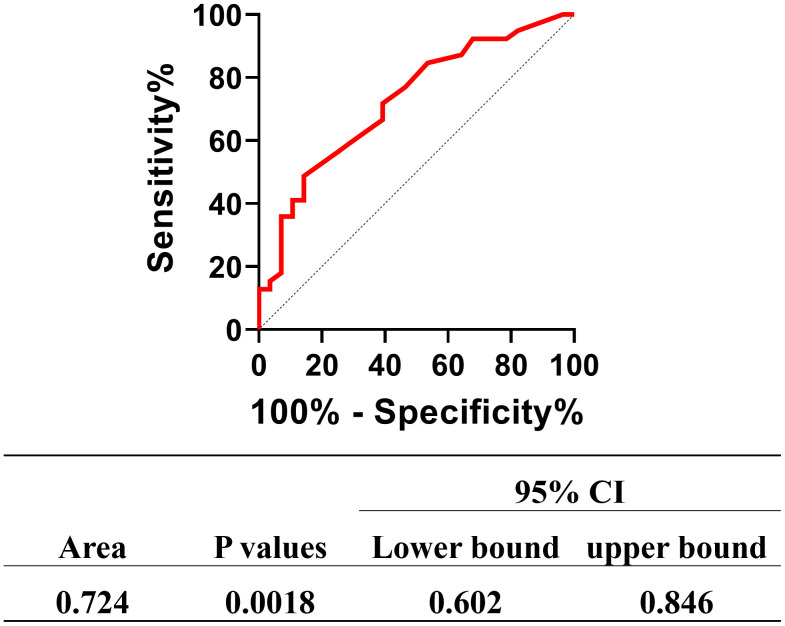
Clinical Predictive Value of Absolute Monocyte Count for Mortality Risk in AIDS/TSM Patients. 53 patients diagnosed with AIDS/TSM were categorized into two groups based on their clinical outcomes: a mortality group (n = 13) and a survival group (n = 40). Based on the absolute counts of two groups, we plotted ROC curves that yielded an AUC of 0.724. Additionally, the baseline absolute monocyte count was found to be a significant predictor of mortality (P = 0.0018).

53 patients diagnosed with AIDS/TSM were categorized into two groups based on their clinical outcomes: a mortality group (n = 13) and a survival group (n = 40). A comparative analysis of baseline clinical data between these groups revealed that the mortality group exhibited significantly lower baseline levels of monocyte count (0.09 ± 0.06 vs 0.26 ± 0.22, *t* = 3.751, *p* < 0.001) than the survival group. Using the absolute counts of two groups to construct ROC curve(shown in Fig 4), we obtained an AUC value of 0.724. Meanwhile, statistical analysis confirmed that the baseline absolute monocyte count was a significant predictor of mortality (*p* = 0.0018).

### 4. AIDS/TSM patients predominantly demonstrate an elevation in classical monocyte levels, accompanied by a reduction in both intermediate and non-classical monocyte populations

**Fig 5:**
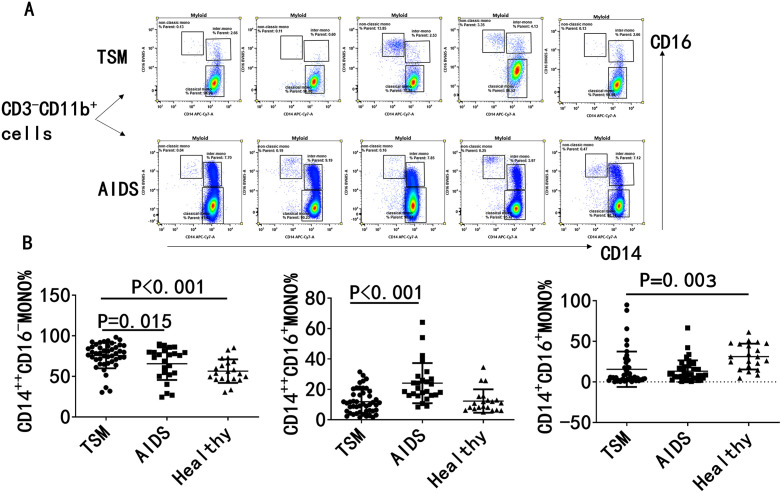
AIDS/TSM patients predominantly demonstrate an elevation in classical monocyte levels, accompanied by a reduction in both intermediate and non-classical monocyte populations. **A** Flow cytometry analysis was conducted on peripheral blood mononuclear cells (PBMCs) derived from patients with AIDS/TSM. From CD3-CD11B+cells, monocytes were classified into classical, intermediate, and non classical types based on the expression of CD14 and CD16. **B** In comparison to the group with uncomplicated AIDS and the healthy control group, the analysis revealed that monocytes in AIDS/TSM patients exhibited an elevation in classical monocytes, alongside a reduction in intermediate and non-classical monocytes.

Flow cytometry analysis was conducted on peripheral blood mononuclear cells (PBMCs) derived from patients with AIDS/TSM. In comparison to the group with uncomplicated AIDS and the healthy control group, the analysis revealed that monocytes in AIDS/TSM patients exhibited an elevation in classical monocytes, alongside a reduction in intermediate and non-classical monocytes, as illustrated in Fig 5.

In addition, the transcriptomic characteristics of monocytes were further analyzed.

Monocytes were isolated from the peripheral blood mononuclear cells (PBMCs) of AIDS/TSM patients and subjected to RNA sequencing. The resulting data were compared with those from a cohort of patients with AIDS alone to identify differentially expressed genes. Subsequent Gene Ontology (GO) and Kyoto Encyclopedia of Genes and Genomes (KEGG) enrichment analyses revealed that monocytes in AIDS/TSM patients exhibited an immune activation status. This immune activation was notably more pronounced in deceased patients. Refer to S1 Fig for detailed visualization.

### 5. Mononuclear cell levels progressively normalize following the administration of antifungal and antiretroviral therapies in AIDS/TSM patients

**Fig 6:**
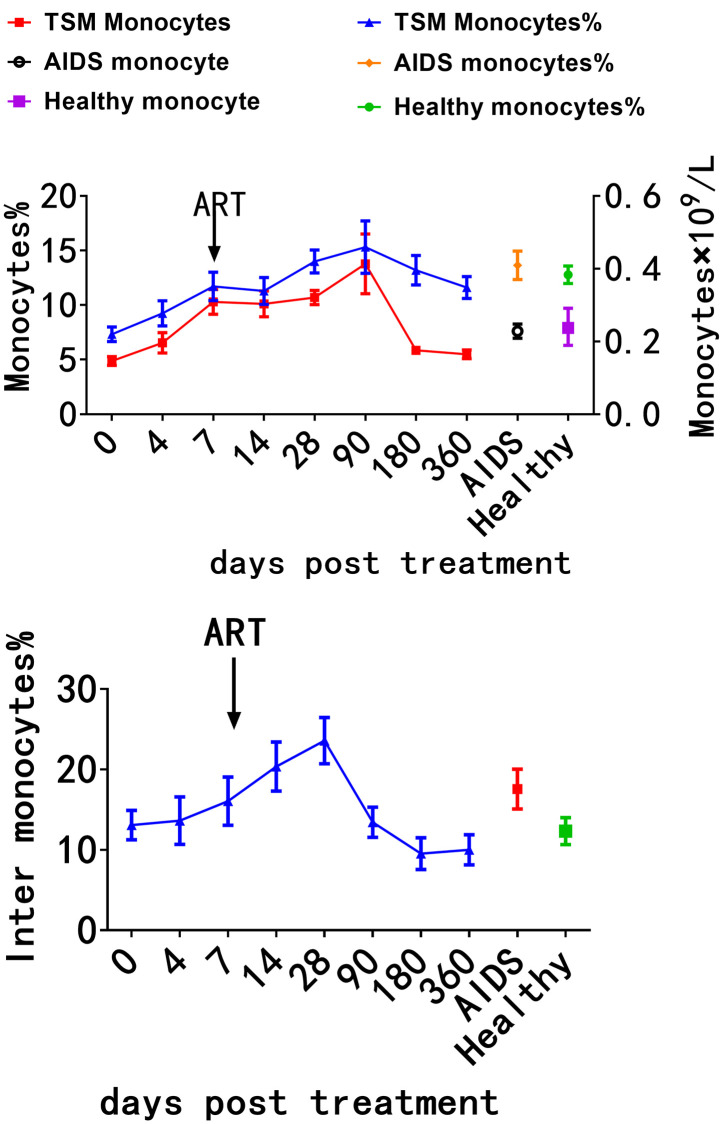
Mononuclear cell levels progressively normalize following the administration of antifungal and antiretroviral therapies in AIDS/TSM patients. AIDS/TSM patients were followed up of after antifungal and antiretroviral therapy. Mononuclear cell levels progressively rise, peaking at 28 weeks, before gradually declining and returning to levels comparable to those of the normal control group after 90 days.

Continue to monitor and follow up with patients surviving AIDS/TSM. Following hospital admission and the commencement of antifungal therapy, antiretroviral therapy (ART) is typically initiated approximately one week later. Mononuclear cell levels progressively rise, peaking at 28 weeks, before gradually declining and returning to levels comparable to those of the normal control group after 90 days. Refer to Fig 6 for further details.

## Discussion

This study systematically analyzed the count, subset distribution, and transcriptomic characteristics of peripheral blood monocytes in patients with AIDS complicated by *Talaromyces marneffei* (TSM) infection. The results demonstrated a significantly decreased total monocyte count, a skewed subset distribution dominated by classical monocytes, and a highly inflammatory activation status in these patients. Moreover, these alterations were closely associated with disease severity and clinical prognosis. Combined with existing immunopathologic evidence, these findings not only deepen the understanding of the pathogenic mechanism of TM in immunocompromised hosts, but also provide potentially valuable immune biomarkers for clinical disease evaluation and therapeutic response monitoring.

As key effector cells of innate immunity, monocytes play a crucial role in fungal recognition, inflammatory recruitment, and host defense. A classic mechanism underlying reduced monocyte counts during acute infection is the rapid mobilization and migration of peripheral blood monocytes toward infected tissues. The significant monocytopenia observed in AIDS/TSM patients in this study is highly consistent with the rapid depletion of monocytes in acute inflammatory models such as endotoxemia and severe viral infections [9], suggesting that TM infection triggers robust innate immune activation and drives directional migration of monocytes to inflammatory lesions. Meanwhile, HIV-induced suppression of bone marrow hematopoietic function directly impairs the regeneration and replenishment of peripheral monocytes, further exacerbating the imbalance between cell consumption and production, ultimately resulting in persistent reduction of circulating monocytes. This dual mechanism—enhanced tissue mobilization and insufficient bone marrow compensation—constitutes the core pathophysiological basis of monocytopenia in AIDS/TSM patients and explains the clinical characteristics of frequent fungal dissemination and rapid disease progression in this population.

The core findings of this study demonstrate that the reduction in peripheral blood monocyte counts is significantly more pronounced in patients with Talaromyces marneffei infection (TSM) compared to those with other common opportunistic infections in AIDS, including classic comorbidities like tuberculosis and Pneumocystis jirovecii pneumonia. This result suggests a more intense systemic inflammatory response in patients with TSM. This finding can reasonably explain why TSM patients are more prone to severe clinical manifestations such as persistent high fever and rapidly progressive multiple organ dysfunction compared to patients with tuberculosis or Pneumocystis jirovecii pneumonia, providing an important pathological basis for understanding the mechanism of severe disease progression in TSM [3].

Imbalanced monocyte subsets represents another important finding of this study. Patients showed a predominant proportion of classical monocytes with relatively reduced intermediate and non-classical monocytes, indicating a highly biased stress pattern of immune response. As the main anti-infective subset, the relative elevation of classical monocytes essentially reflects a compensatory host response against invasive fungal infection. However, in the context of severe CD4 + T-cell deficiency, such compensation fails to translate into effective pathogen clearance, and may instead aggravate tissue damage due to sustained and uncontrolled inflammatory activation. This subset profile is similar to reports in severe COVID-19 and acute myocardial infarction, suggesting that severe infection-driven dominance of classical monocytes may represent a common immune phenotype in critical inflammatory states [10,11]. The immune dysfunction accompanying subset imbalance may further impair pathogen elimination, amplify inflammatory cascades, and thereby promote disease deterioration.

Transcriptomic results further confirmed that monocytes in AIDS/TSM patients exhibited significant inflammatory activation, which was even more pronounced in non-survivors, indicating that the intensity of monocyte inflammatory activation may serve as an important prognostic indicator. In the absence of effective adaptive immune support, inflammatory mediators released by overactivated monocytes are poorly regulated, predisposing to a vicious cycle of inflammation–tissue injury–immune exhaustion. This shares mechanistic similarities with severe COVID-19, in which pyroptosis, metabolic reprogramming, and pro-inflammatory monocyte phenotypes drive systemic inflammation [12,13], suggesting that in severe immunodeficiency or critical infection, monocytes act not only as key defenders but also important mediators of inflammatory injury. The degree of functional imbalance directly determines whether the host can achieve a balance between pathogen clearance and inflammation control.

Following combined antifungal therapy and ART, monocyte counts gradually recovered and subset distribution tended to normalize, indicating progressive restoration of immune function along with pathogen control. The kinetic recovery of monocytes post-treatment coincided with reduced inflammation and suppressed HIV RNA levels, demonstrating that normalization of monocyte count and subsets can serve as a dynamic indicator for immune function repair and improved clinical outcomes. In various diseases including first-episode psychosis [14], cardiac surgery [15], and COVID-19 [12], the recovery of monocyte levels has been regarded as a marker of disease remission and immune homeostasis, further supporting its potential value as a broad-spectrum prognostic biomarker.

A recent review described the inflammatory hyperresponse during TM infection. The core viewpoint of the article is as follows: The pathogenesis of TM infection is closely associated with the diverse intensity spectrum of the host’s immune system, and it regulates the occurrence and severity of the disease [16]. This study is consistent with the core viewpoint of the review. The monocytes from TM patients are in a state of extreme activation and exhaustion, which explains that the mononuclear phagocyte system is an important mechanism for AIDS patients to resist TM infection. Meanwhile, the excessive immune response constitutes the pathogenic mechanism of severe illness and death.

In summary, this study systematically revealed characteristic monocyte alterations and their clinical significance in AIDS/TSM patients at the cellular, subset, and molecular phenotypic levels. The rapid mobilization of monocytes induced by TM infection, insufficient replenishment due to HIV-related bone marrow hematopoietic injury, immune imbalance mediated by subset skewing, and tissue injury driven by excessive inflammatory activation collectively constitute important immune mechanisms underlying disease progression. Monocytes are not only involved in host defense but also serve as a key node linking inflammatory injury and prognostic evaluation.

Nevertheless, the study has certain limitations. For instance, while we observed that a reduction in monocyte count may impact disease prognosis, this finding has yet to be validated in larger populations, and there is a dearth of research on prognostic models. Additionally, although a decrease in monocyte count has been associated with inflammation activation, research on specific pathways and mechanisms remains insufficient. Thirdly, the influence of confounding factors was not fully controlled, which is an important limitation of this study. On the one hand, there may be heterogeneity in treatment regimens, and different antifungal drugs (such as azoles and echinocandins) and ART drugs may have different effects on the count, subset distribution, and functional status of monocytes, thereby interfering with the accuracy of the study results. On the other hand, concurrent medications and underlying diseases were not included in the analysis. Some patients may have underlying diseases such as hypertension, diabetes mellitus, and chronic liver disease, and all these factors may independently or synergistically affect the level of peripheral blood monocytes, thus affecting the rigor of the study conclusions.

Limited by the single-center design and sample size, this study did not perform in-depth functional validation of key pathways and molecular mechanisms. In the future, single-cell sequencing, in vitro functional assays, and animal models may be used to further clarify the specific mechanisms of monocyte polarization, metabolic reprogramming, and interaction with fungi, providing new theoretical evidence for developing targeted immunomodulatory strategies and improving the prognosis of AIDS patients with deep fungal infections.

## Supporting information

S1 Fig Mononuclear cells in patients with AIDS/TSM demonstrate characteristics of hyperactivation. Monocytes were isolated from the peripheral blood mononuclear cells (PBMCs) of AIDS/TSM patients and subjected to RNA sequencing. A Volcanic map of differentially expressed genes between AIDS/TSM and simple AIDS patients; B The distribution of differentially expressed genes between AIDS/TSM and simple AIDS patients in survied and deceased groups; C. Differential expression gene GO and KEGG analysis of CD14 monocytes between AIDS/TSM patients and simple AIDS patients.
(TIF)

S1 Table The baseline clinical indicators of deceased and survived groups in AIDS/TSM patients. (TIF)
