# Supplementary figures and images for: AIDS patients with Talaromycosis Marneffei exhibit inflammatory activation and depletion in their peripheral blood monocytes
Source: PLoS Negl Trop Dis. 2026 May 4;20(5):e0014306. doi: 10.1371/journal.pntd.0014306 (PMC13167028; doi:10.1371/journal.pntd.0014306)

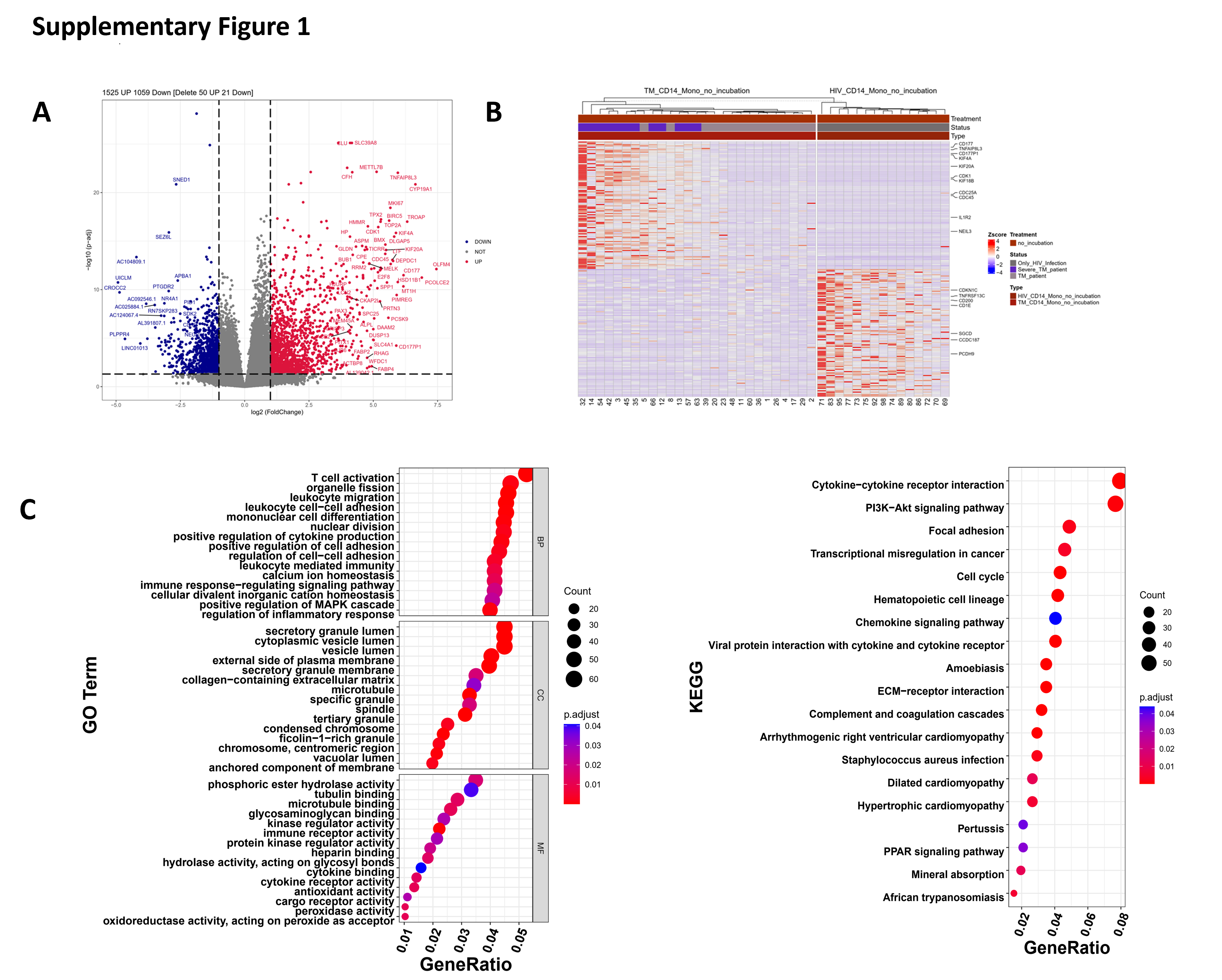

Supplement: S1 Fig — Monocytes were isolated from the peripheral blood mononuclear cells (PBMCs) of AIDS/TSM patients and subjected to RNA sequencing. A Volcanic map of differentially expressed genes between AIDS/TSM and simple AIDS patients; B The distribution of differentially expressed genes between AIDS/TSM and simple AIDS patients in survied and deceased groups; C. Differential expression gene GO and KEGG analysis of CD14 monocytes between AIDS/TSM patients and simple AIDS patients. (TIF) [file pntd.0014306.s001.tif]

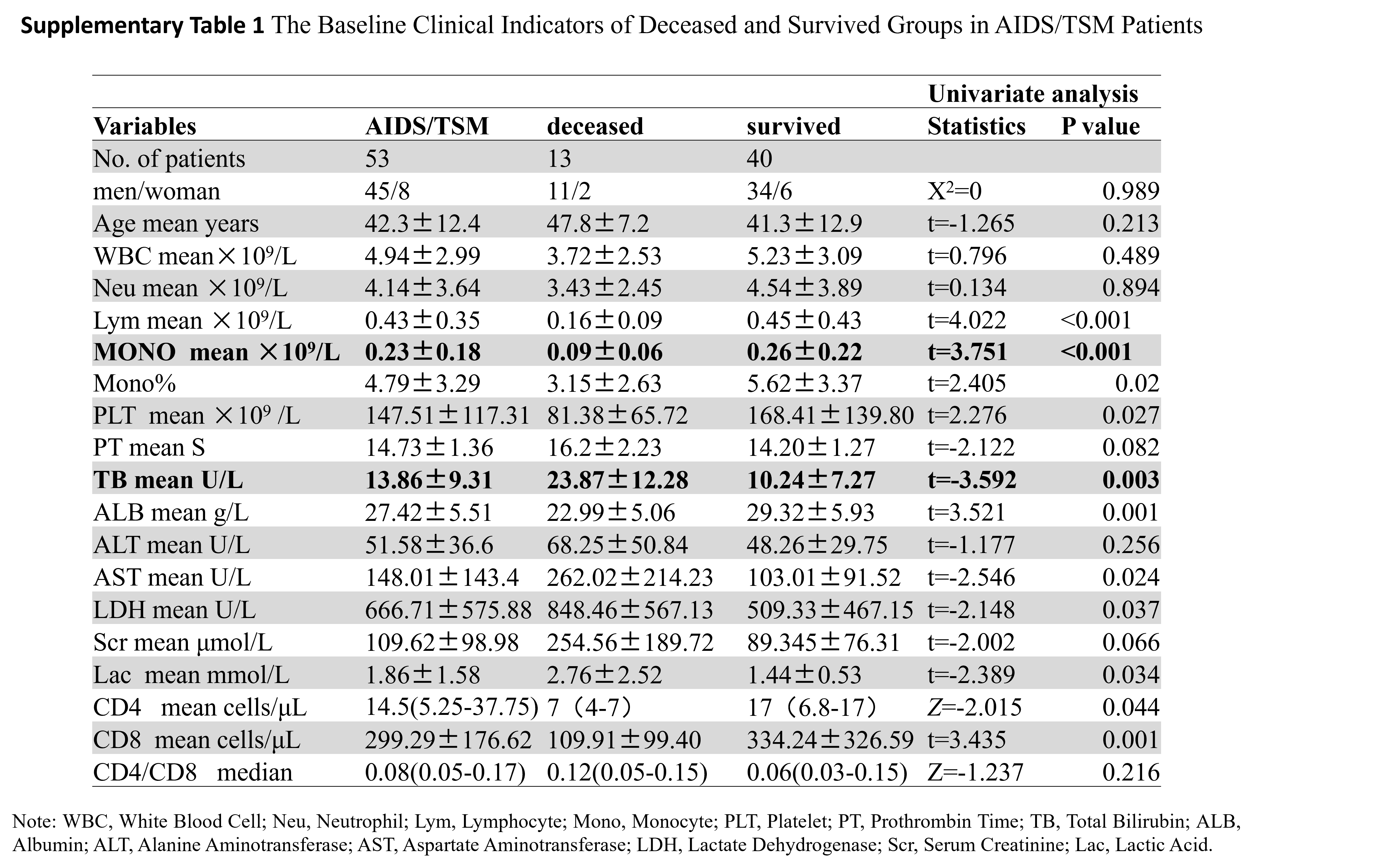

Supplement: S1 Table — (TIF) [file pntd.0014306.s002.tif]
